# TM4SF1 is a molecular facilitator that distributes cargo proteins intracellularly in endothelial cells in support of blood vessel formation

**DOI:** 10.1002/ccs3.12031

**Published:** 2024-05-07

**Authors:** Chi‐Iou Lin, Anne Merley, Shou‐Ching S. Jaminet

**Affiliations:** ^1^ Center for Vascular Biology Research and Department of Pathology Beth Israel Deaconess Medical Center and Harvard Medical School Boston Massachusetts USA; ^2^ Department of Anesthesiology Riverview Hospital Noblesville Indiana USA; ^3^ Center for Animal Resources and Education Brown University Providence Rhode Island USA; ^4^ Biology Department Angiex Inc. Cambridge Massachusetts USA

**Keywords:** angiogenesis, HDAC6, PLCγ, protein trafficking, TM4SF1‐mediated endocytosis

## Abstract

Transmembrane‐4 L‐six family member‐1 (TM4SF1) is an atypical tetraspanin that is highly and selectively expressed in proliferating endothelial cells and plays an essential role in blood vessel development. TM4SF1 forms clusters on the cell surface called TMED (TM4SF1‐enriched microdomains) and recruits other proteins that internalize along with TM4SF1 via microtubules to intracellular locations including the nucleus. We report here that tumor growth and wound healing are inhibited in *Tm4sf1*‐heterozygous mice. Investigating the mechanisms of TM4SF1 activity, we show that 12 out of 18 signaling molecules examined are recruited to TMED on the surface of cultured human umbilical vein endothelial cells (HUVEC) and internalize along with TMED; notable among them are PLCγ and HDAC6. When TM4SF1 is knocked down in HUVEC, microtubules are heavily acetylated despite normal levels of HDAC6 protein, and, despite normal levels of VEGFR2, are unable to proliferate. Together, our studies indicate that pathological angiogenesis is inhibited when levels of TM4SF1 are reduced as in *Tm4sf1*‐heterozygous mice; a likely mechanism is that TM4SF1 regulates the intracellular distribution of signaling molecules necessary for endothelial cell proliferation and migration.

## Graphical abstract

TM4SF1‐enriched microdomain (TMED) are transported along microtubules to the microtubule organization center (MTOC) and then enter the nucleus via nuclear pores where TMED are being processed. This internalization mechanism delivers TMED‐recruited cargo molecules for cell signaling in actively proliferating and mobile endothelial cells.

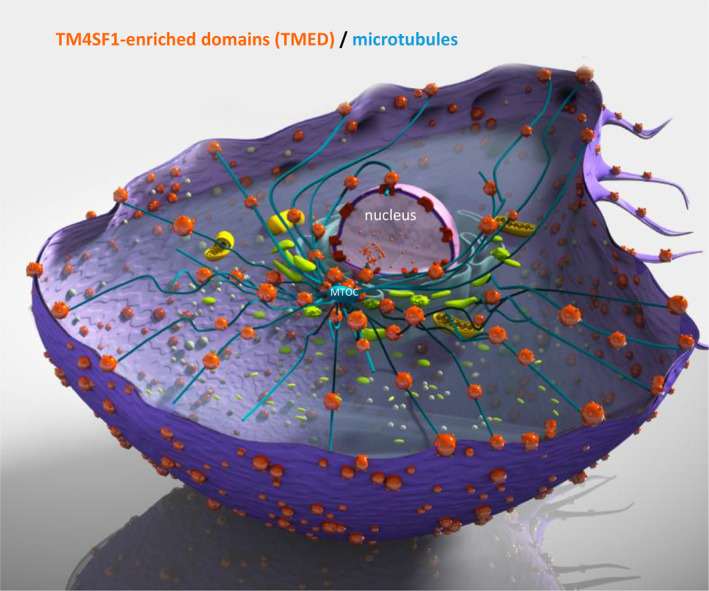

## 1 INTRODUCTION

Transmembrane‐4 L‐six family member‐1 (TM4SF1) is a small plasma membrane glycoprotein with tetraspanin topology but not homology.^1^ TM4SF1 was known to be a tumor cell‐associated antigen expressed at low levels in normal vascular endothelium.^2^, ^3^, ^4^, ^5^ We demonstrated that TM4SF1 is expressed at high levels in vivo in vascular endothelium at sites of pathological angiogenesis, both in mice^6^, ^7^ and in human cancers,^7^, ^8^ and also in vitro in proliferating blood and lymphatic endothelial cells and mesenchymal stem cells.^9^, ^10^ We further demonstrated that TM4SF1 is critical for developmental vasculogenesis: *Tm4sf1*‐null C57BL/6 mice are avascular and embryonically lethal at E9.5. Further, almost half of *Tm4sf1*‐heterozygous mice die in utero by E17 due to intraventricular brain hemorrhage^10^ but the majority that survive develop normally, with no increased post‐natal mortality or morbidity.

TM4SF1 forms 100–300 nm diameter protein clusters on the cell surface called TMED (TM4SF1‐enriched microdomains).^7^, ^8^, ^11^ Each TMED contains 3 to 14 TM4SF1 molecules and TMED increase in number and size when TM4SF1 expression is high, such as in proliferating endothelial cells in vitro^7^, ^8^, ^11^ and in tumor endothelium in vivo^8^ TMEDs function as membrane docks that recruit a variety of other membrane proteins and their intracellular membrane‐proximal signaling components.^8^, ^11^ Therefore, TM4SF1 shares this microdomain‐forming and protein‐complex‐recruiting ability with genuine tetraspanins.^12^, ^13^

TMEDs internalize via microtubules whose ultimate destination is the nuclear compartment.^8^ This novel internalization mechanism differs from that of many genuine tetraspanins, which contain adapter protein motifs and internalize via clathrin‐mediated endocytosis and whose ultimate destination is endosomes‐lysosomes or exosomes.^12^, ^13^ TM4SF1 lacks any known internalization motifs recognized by clathrin adapter proteins^13^, ^14^ and clathrin inhibitors do not block TMED internalization.^8^ Depletion of TM4SF1 through knockdown in cultured endothelial cells in vitro prevents the cellular polarization necessary for migration and proliferation, and inhibits EC‐EC interactions for junction formation,^7^, ^11^ and, in a surrogate skin angiogenesis model in vivo, inhibits vessel maturation.^7^

In this study, we first investigated TM4SF1's ability to support pathological angiogenesis for tumor growth and wound healing in *Tm4sf1*‐heterozygous mice. Then, pursuing the mechanism by which TM4SF1 acted, we sought to identify the cargo molecules recruited to TMED in support of endothelial cell migration and proliferation. We now report (i) inhibition of B16F10 tumor growth and wound healing in *Tm4sf1*‐heterozygous mice, and (ii) that 12 out of 18 signaling molecules examined are recruited to TMED on the cell surface and 10 were found to internalize with TMED; PLCγ and HDAC6 notable among them. Also, when TM4SF1 was knocked down in human umbilical vein endothelial cells (HUVEC), microtubules were heavily acetylated despite normal levels of HDAC6 protein, and endothelial cell proliferation was inhibited despite normal levels of VEGFR2. Together, our studies indicate that TMEDs act as vehicles for the proper intracellular distribution of cargo molecules essential for endothelial cell proliferation and migration in vivo and in vitro.

## 2 MATERIALS AND METHODS

### 2.1 Antibodies and reagents

Anti‐human TM4SF1 antibody (Ab) 8G4 was the same as used in our earlier studies. (i) m8G4: 8G4 mouse monoclonal IgG1^9^ (Millipore Sigma catalog number MABC1723) and was employed in Western blot; and (ii) h8G4: 8G4 antibody with the constant region of a human IgG1^15^ and was employed in cell staining, immunoprecipitation (IP), reverse IP and Western blot. A combination of h8G4 IP and m8G4 Western blot was used to eliminate 2nd antibody‐initiated IgG cross reactivity; in the Western blot, m8G4 interacts with TM4SF1 that was pulled down by h8G4, and anti‐mouse 2nd antibodies do not interact with the hIgG1 heavy and light chains from h8G4 in the SDS‐PAGE. Details of all primary and secondary antibodies used in this study, including the isotype matched control antibodies, are summarized in Supplemental Table S1. Phalloidin‐TRIC and mouse IgG1 were purchased from was purchased from ThermoFisher (Waltham, MA).

### 2.2 Cells and cell culture

HUVEC (human umbilical vein EC) was purchased along with its respective media from Lonza (Walkersville, MD), and used within passage six in all experiments conducted in this study. B16F10 mouse melanoma cell line syngeneic to C57BL/6 mice were acquired from ATCC (American Type Culture Collection), cultured in RPMI (10% FBS), and was used within five passages after being awakened from liquid nitrogen.

### 2.3 TM4SF1 knockdown in HUVEC

Short hairpin RNA (shRNA) adenoviruses for control (GTACGTACGTACGTACGTACT) and TM4SF1 knockdown (KD; GCACGATGCATCGGACATTCT) were described previously.^7^ HUVECs grown in complete media at ∼30% confluency was transduced with 25 moi of either control or TM4SF1‐KD adenoviruses for three days to achieve ≥90% reduction of TM4SF1 mRNA expression.^7^ Cells were either fixed in 4% paraformaldehyde for cell staining or protein extraction for Western blot.^16^

### 2.4 Tm4SF1‐heterozygous mice

*Tm4sf1*‐heterzygous male and female mice (stabilized in C57BL/6 background)^10^ were mated to obtain littermates of *Tm4sf1*‐heterzygous and ‐wild type mice for the study. Genotyping was conducted using chromosomal DNA extracted from 2 mm tail clip from P14 to P21 postnatal mice using the PCR primers that are specific for the wild‐type and targeted alleles as described previously.^10^ Both wild type and *Tm4sf1*‐heterozygous share forward primers (5′CAGACTGGAAACGGTCCAAAAGGCTGCAAG3′) with distinctive different reverse primers to distinguish wild type (5′GAAACGGCTGAGGTGGTCCTCCGTAGCATAC3′) from *Tm4sf1* target (5′CTGGTTGCTGACTAATTGAGATGCATGCTTTGC3′) allele and respectively generates 596 bp PCR fragment and 397 bp PCR fragment from targeted alleles on 1% agarose gels.

### 2.5 Cutaneous mouse ear wound healing model and B16F10 syngeneic mouse tumor growth model

Seven to eight‐week‐old *Tm4sf1*‐wild type and ‐heterozygous mice (C57BL/6 background^10^) were used in both wound healing and B16F10 syngeneic tumor models. Animals were housed and handled according to institution‐approved animal care protocols.

For the cutaneous mouse ear wound healing, mice were anaesthetized under 1%–2% isoflurane, sterilized mouse ear using Providone iodine swab stick (Dynarex, Orangeburg, NY), and made a 4 mm biopsy punch wound in the center of the cartilaginous region of each ear through a disposable biopsy punch (Integra LifeSciences). The ears were imaged post‐injury at day‐0, ‐7, ‐14, ‐21, and ‐28 using a live cam attached Wild Photo Mikroskop M400 stereo photomicroscope (Martin Microscope) while mice were anaesthetized under the 1%–2% isoflurane. Wound closure in the ear images was quantified by measuring the surface area of the hole using ImageJ (NIH) and calculating percent difference compared with original ear punch area.

For the B16F10 syngeneic mouse tumor growth model, mice were inoculated subcutaneously with 1 million B16F10 tumor cells at day‐0 and measured tumor volume every six days.

### 2.6 Immunoprecipitation and Western blot

Experimental procedures were described in detail previously.^11^ For TM4SF1 pull‐down IP assays, HUVEC were grown in complete media to 80%–90% confluency in 15 cm culture plates, trypsinized to suspension in TBS (10^7^ cells/100 μl, pH 7.0), and incubated with either 5 μg h8G4 or isotype matched control hIgG1 for 30 min on ice. Unbound antibodies were removed by washing in cold TBS for 2 times with centrifugation for 5 min in 4°C in between the wash, and the cell pellets were either (i) directly extracted proteins by incubating for 30 min on ice after suspending in 200 μl cold TBS containing protease/phosphatase inhibitor cocktails (Millipore Sigma) with varying concentration of Triton X‐100 (ThermoFisher), or (ii) returned to culture in a 10 cm culture plate with a complete medium at 37°C for 2 h before the protein extraction. Protein extracts were gathered after centrifugation in 12,000 rpm for 10 min at 4°C and added Protein‐G Magnetic Dynabeads (ThermoFisher) to pull down IgGs followed by washing in 1 mL cold TBS containing protease/phosphatase inhibitor cocktails for SDS‐PAGE. Western blot with appropriate primary and HRP (horseradish peroxidase)–conjugated secondary antibodies (ThermoFisher). A 2nd Ab against the heavy chain of the antibodies used in IP was applied for monitoring experiments and sample loading in SDS‐PAGE. Blots were developed with the ECL system (GE Healthcare Life Sciences). All Western blot images were representative selections from at least three separate experiments.

### 2.7 Immunocytochemical staining

Experimental procedures were described in detail previously.^16^ Briefly, cells were grown on glass disc (ThermoFisher), fixed with 4% paraformaldehyde, washed in PBS, and blocked with PBS/2% FBS prior to immunostaining with primary antibodies 8G4 (mouse anti‐human TM4SF1, IgG1 isotype)^8^ or rat anti‐human *α*‐tubulin (Santa Cruz Biotechnology), followed by secondary donkey anti‐mouse (or anti‐rat) Alexa Fluor‐488 or ‐594 labeled antibodies (Life technology). Nikon TE‐300 was used to capture epifluorescence images. All immunocytochemistry images were representative selections from at least three separate experiments.

### 2.8 Statistical analysis

Each in vivo experiment was repeated at least three times and the statistical difference between groups is presented as mean ± standard deviation. Analysis was performed using GraphPad Prism 10.0 software. The significance of differences between groups was assessed by Student's *t*‐test; *p* values < 0.05 were considered statistically significant.

## 3 RESULTS

### 3.1 Impaired tumor growth and wound healing in Tm4sf1‐heterozygous (+/−) mice

**FIGURE 1:**
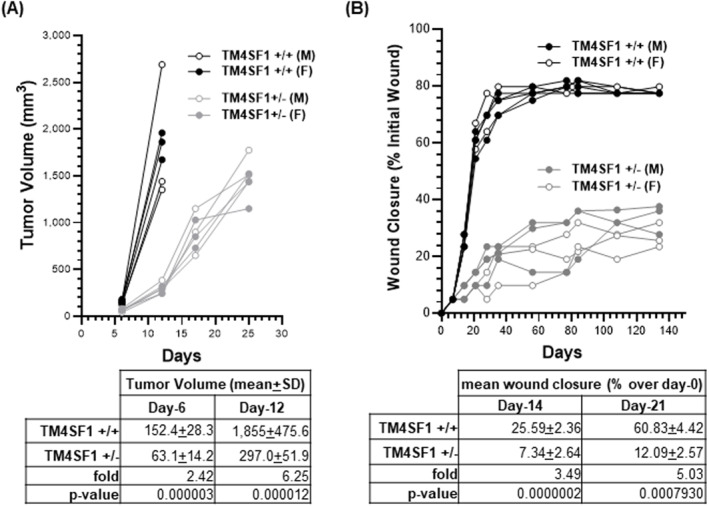
Tumor growth and wound healing are hindered in *Tm4sf1*‐heterozygous (+/−) mice. (A) B16F10 melanoma tumor growth and (B) cutaneous mouse ear wound closure models were studied in both 8‐week‐old *Tm4sf1*‐wild type (+/+) and ‐heterozygous (+/−) mice, with each study group composed of three males (M) and three females (F) of littermates, and each experiment was repeated minimal three times. Representative data are presented in both (A, B). (A) Slower B16F10 mouse melanoma tumor growth in *Tm4sf1* +/− mice. Mean tumor volume at day‐6 was 152.4 ± 18.3 mm^3^ for the +/+ in comparison to 63.1 ± 14.2 mm^3^ for the +/−, a 2.4‐fold difference (*p* = 0.000003, Student's *t*‐test). At day‐12, +/+ group had attained nearly 10% of mouse body weight with a mean tumor volume of 1855 ± 475.6 mm^3^, while +/− group had reached a 297 ± 51.9 mm^3^, a 6.25‐fold difference (*p* = 0.000012, Student's *t*‐test); mean tumor volume in the +/− mice was reached to 1472 ± 200.9 mm^3^ on day‐25. (B) Impaired wound healing in *Tm4sf1* +/− mice. Wound closure was quantified by measuring the surface area of each ear wound image via ImageJ software, after which a percent closure relative to the initial wound size (4 mm in diameter) was calculated. *Tm4sf1* +/+ mice exhibited linear wound closure in the first 21 days and achieved 25.59 ± 2.36% and 60.83 ± 4.42% closure at day‐14 and day‐21, respectively. *Tm4sf1* +/− mice exhibited 7.34 ± 2.64% and 12.09 ± 2.57% closure compared to the initial wound at day‐14 and day‐21, respectively. This signifies 3.49‐fold (day‐14; *p* = 0.0000002 Student's *t*‐test) and 5.03‐fold (day‐21; *p* = 0.0007930, Student's *t*‐test) greater wound closure in wild type than in *Tm4sf1*‐heterozygous mice. Wound closure entered stationary phase near day‐28. Similar outcomes were seen in both male and female littermate mice in both tumor and wound healing models.

Two models, the B16F10 melanoma tumor model syngeneic to the C57BL/6 mouse strain (Figure 1A) and a cutaneous mouse ear wound model (Figure 1B), were employed to study the effects of TM4SF1 in tumor growth and wound healing using *Tm4sf1* +/− mice. In *Tm4sf1* wild type mice, mean tumor volumes were 152.4 ± 28.3 mm^3^ at day‐6 and 1855 ± 475.6 mm^3^ at day‐12, after which mice had to be sacrificed (Figure 1A). In contrast, mean tumor volumes in *Tm4sf1*‐heterozygous mice were 63.1 ± 14.2 mm^3^ at day‐6 and 297 ± 51.9 mm^3^ at day‐12, and tumors ultimately reached a size of 1472 ± 200.9 mm^3^ on day‐25 (Figure 1A). Thus, tumor volumes in wild type mice were 2.42‐fold (day‐6; *p* = 0.000003 Student's *t*‐test) and 6.25‐fold (day‐12; *p* = 0.000012, Student's *t*‐test) higher than in *Tm4sf1*‐heterozygous mice; similar results were obtained in both male and female mice (Figure 1A).

A cutaneous ear wound healing model presented a comparable outcome. In wild type mice, ear wound diameter decreased from an initial 4 mm in diameter to about 2 mm over the course of 28 days, following which residual ear wound size stabilized over the remaining 134 days of the tracking period (Figure 1B). Mean wound surface area decreased by 25.59% ± 2.36% at day‐14% and 60.83% ± 4.42% at day‐21 in wild type mice. In contrast, in *Tm4sf1*‐heterozygous mice, ear wound healing was significantly slower; ear wound area decreased only 7.34% ± 2.64% by day‐14% and 12.09% ± 2.57% by day‐21 (Figure 1B). This represents 3.49‐fold (day‐14; *p* = 0.0000002, Student's *t*‐test) and 5.03‐fold (day‐21; *p* = 0.0007930 Student's *t*‐test) greater wound closure in wild type than in *Tm4sf1*‐heterozygous mice; male and female mice showed similar wound healing outcomes (Figure 1B).

### 3.2 TM4SF1‐enriched microdomains transport cargo molecules to intracellular destinations in endothelial cells

We next investigated the mechanisms by which TM4SF1 functions in cultured endothelial cells. We have shown previously that TM4SF1 form clusters called TMED on the cell surface that these TMED include a number of other molecules^7^, ^11^ in addition to TM4SF1, including αTubulin^8^; also, TMED internalize constitutively from the cell surface along microtubules to enter the cell nucleus in cultured endothelial cells^8^ (illustrated in Supplementary Figure 1). We now asked whether TMED carried molecules and delivered them to specific intracellular destinations; such functions have been reported for genuine tetraspanins which internalize via a clathrin‐mediated pathway.^13^ To investigate this question, we used IP and Western blot analysis in HUVEC. Previously we had shown that TMED are highly sensitive to Triton X‐100 and a brief 30‐min treatment of HUVEC with 0.05% Triton X‐100 at 4–8°C removes nearly 90% of TMED from the cell surface but spares TMED in the perinuclear region; 0.1% Triton X‐100 removes nearly all TMED including internalized TMED in the perinuclear region (see representative results in Supplementary Figure 2).^7^, ^11^ The distinct sensitivities of cell surface and perinuclear TMED to Triton X‐100 is advantageous for IP‐Western analysis for the identification of TMED‐recruited proteins and their internalization, as cell surface and internalizing TMED‐Ab complexes can be extracted with 0.05% Triton X‐100 and then subsequent treatment of the cells with 0.1% Triton X‐100 isolates remaining internalized TMED in the perinuclear region.

Three TM4SF1 proteins formed by post‐translational modifications, namely 22kD, 25kD, and 28kD, can be identified in HUVEC Triton X‐100 extracts by Western blots using the m8G4 antibody (Supplementary Figure 2B).^11^ The 28kD TM4SF1, which is now known to be phosphorylated in tyrosine (Supplementary Figure 2C), is the predominant form on the cell surface and is the only form that internalizes and enters the nuclear compartment.^8^

**FIGURE 2:**
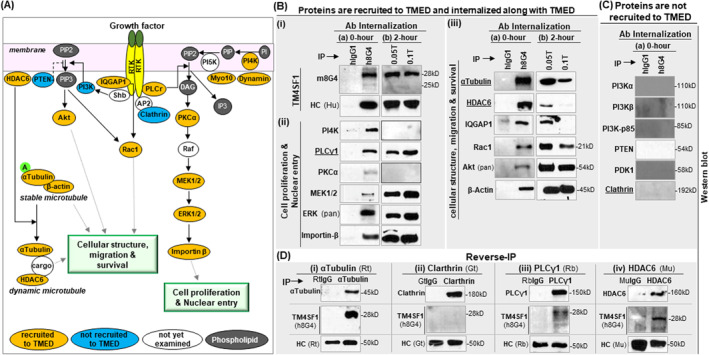
TM4SF1 recruits and transports signaling molecules involved in HUVEC proliferation and migration. (A) Schematic illustration of representative signaling molecules associated with cell migration and proliferation in endothelial cells. Gray fills represent signaling phospholipid. Orange, blue, and white fills respectively indicate the proteins are found on TMED, are not found on TMED, and have not yet been examined in this study. (B) Immunoprecipitation (IP) were performed with hIgG1 (control) or h8G4 using the same approach described in Figure S1,C; Western blots were conducted with m8G4 to identify TM4SF1 (B,i) or with suitable antibodies to identify proteins involved in either cell proliferation and nuclear entry (B,ii), or cellular structure, migration and survival (B,iii). Proteins that failed to show positive bands in the Western were grouped in (C). All experiments were repeated minimal three times. Representative IP‐Western from the h8G4 pull‐down on cell surface at 0‐h h8G4 internalization showed (B,i) 28kD TM4SF1 along with (B,ii) PI4K, PLCγ1, PKCα, MEK1/2, ERK (pan) and Importin‐*β*, and (B,iii) αTubulin, HDAC6, IQGAP1, Rac1, Akt (pan), and *β*‐Actin, but not (C) PI3Kα, PI3Kβ, PI3Kp85, PTEN, PDK1, and Clathrin. Isotype matched control antibody, hIgG1, did not bind to the cell as evidenced by no detection of heavy chain (HC) (B,i) along with lack of any positive protein bands in the Western blots (B,i,ii,iii). Other than PI4K and PKCα, all proteins showing positive interactions with TM4SF1 on cell surface before internalization at 0‐h continuously showed positive protein bands after the 2‐h internalization. Extraction with 0.1% Triton X‐100 after an initial 0.05% Triton X‐100 treatment enabled TMED extraction by the perinuclear. (D) Four proteins, (i) αTubulin, (ii) clathrin, (iii) PLCγ1, and (iv) HDAC6 were subjected to reverse IPs in the total HUVEC cell lysate (underlined in Bi,ii,iii and C); h8G4 was employed in the Western to avoid IgG cross reactivity with the antibodies used in IP. Clathrin (negative control) IP pull‐down did not identify TM4SF1, while PLCγ1, HDAC6 and αTubulin (positive control) positively pulled‐down 28kD TM4SF1 in the Western. HC (Hu; Rt; Gt; Mu), HC (Human; Rat; Goat; Murin); 2nd Ab against the HC of the antibodies used in IP was applied to monitor antibody loading in SDS‐PAGE. HUVEC, human umbilical vein endothelial cells.

Figure 2A lists a number of signaling proteins that are known to be important in endothelial cell proliferation and migration through receptor tyrosine kinase (RTK) activation.^17^, ^18^ We made use of 18 different antibodies (Supplementary Table 1) along with anti‐human TM4SF1 antibodies to perform IP‐Western analysis (Figure 2B). Previously, we demonstrated that TM4SF1 interacts with αTubulin but not with clathrin in internalization^8^; thus antibodies against αTubulin and clathrin were employed to respectively serve as positive and negative controls of the IP‐Western (Figure 2B,C).

**FIGURE 3:**
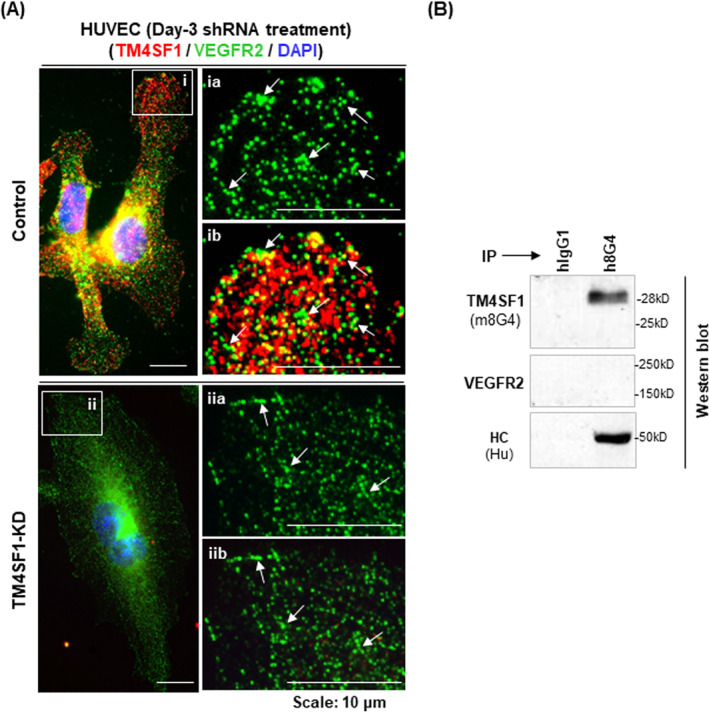
VEGFR2 is not recruited to TMED in HUVEC. (A) Immunocytochemical (ICC) co‐staining of TM4SF1 and VEGFR2 was performed using 3‐day control or TM4SF1‐knockdown (KD) short hairpin RNA adenovirus transduced HUVECs. A representative ICC image shows that TM4SF1 and VEGFR2 were spatially adjacent to each other but lacked overlapping in their distributions (A,i,a,b, white arrows). VEGFR2 staining and cellular distribution were not affected by the enlarged and frequently binuclear TM4SF1‐KD cells (A,ii,a,b, white arrows). (B) Representative IP‐Western showed that h8G4 immunoprecipitation pull‐down contained TM4SF1 but not VEGFR2. Heavy chain (Hu), 2nd Ab anti‐human IgG to monitor h8G4 level in Western blot. HUVEC, human umbilical vein endothelial cells.

Cell surface TMED‐h8G4 pull‐down (0‐h internalization; Figure 2B) extracted 28kD TM4SF1 and 12 out of 18 other proteins examined, as shown by representative IP‐Western. Among those, five proteins are known to be involved in cell proliferation (Figure 2B,ii; PI4K, PLCγ1, PKCα, MEK1/2, and ERK), one enables nuclear internalization (Figure 2B,ii; importin‐β), and six proteins are important for cellular structure, migration and cell survival (Figure 2B,iii; αTubulin, HDAC6, IQGAP1, Rac1, Akt, and *β*‐actin). Five proteins (PI3Kα, PI3Kβ, PI3Kp85, PTEN, and PDK1), along with clathrin, did not show protein bands in Western blots from the TMED‐h8G4 pull‐down (Figure 3C).

To investigate whether any of the proteins recruited to TMED on the cell surface were also present on internalized TMED, we returned h8G4 pre‐labeled HUVEC to culture for 2 h (2‐h internalization; Figure 2B) and then performed sequential extraction with 0.05% and 0.1% Triton X‐100 for IP‐Westerns. Other than PI4K and PKCα, all proteins that were recruited to TMED at 0‐h were also present at 2‐h IP‐Western (Figure 2B); PI4K and PKCα were no longer present on internalized TMED at 2‐h, indicating a transient association only. As expected, 0.1% Triton X‐100 extracted more TM4SF1 (Figure 2B,i) as well as more of the recruited proteins (Figure 2B,ii,iii).

PLCγ1 is the key signaling mediator downstream to receptor tyrosine kinases and is critically involved in endothelial cell proliferation^19^ while HDAC6 is a cytosolic deacetylase and critically regulates cell motility and apoptosis.^20^ Along with αTubulin (positive control; Figure 2D,i) and clathrin (negative control; Figure 2D,ii), we performed reverse IP for PLCγ1 (Figure 2D,iii) and HDAC6 (Figure 2D,iv) from the HUVEC cell lysate. As expected, clathrin IP did not pull down TM4SF1; also, as expected, the 28kD TM4SF1 protein was detected in the IPs from αTubulin, PLCγ1 and HDAC6. This further demonstrates that PLCγ1 and HDAC6 interact with TM4SF1.

### 3.3 TMED influences endothelial cell function through recruited cargo molecules

PLCγ1 is a direct downstream effector molecule of VEGFR2 activation.^19^ Hence, we investigated whether the association of PLCγ1 with TMED is linked to the recruitment of VEGFR2 to TMED (Figure 3). Immunofluorescence studies demonstrated that TM4SF1 and VEGFR2 did not co‐localize (Figure 3A,i,a,b; white arrows); moreover, the cellular distribution and the relative abundance of VEGFR2 protein in the enlarged bi‐nuclear TM4SF1‐knockdown HUVEC resembled that in control HUVEC (Figure 3A,ii,a,b; white arrows). Representative IP‐Westerns demonstrated that VEGFR2 protein was absent from the h8G4 antibody‐driven TM4SF1 pull‐down extracts (Figure 3B,i). Our study demonstrates that VEGFR2's downstream signaling cascade is inhibited when TM4SF1 is depleted in knockdown HUVEC.

**FIGURE 4:**
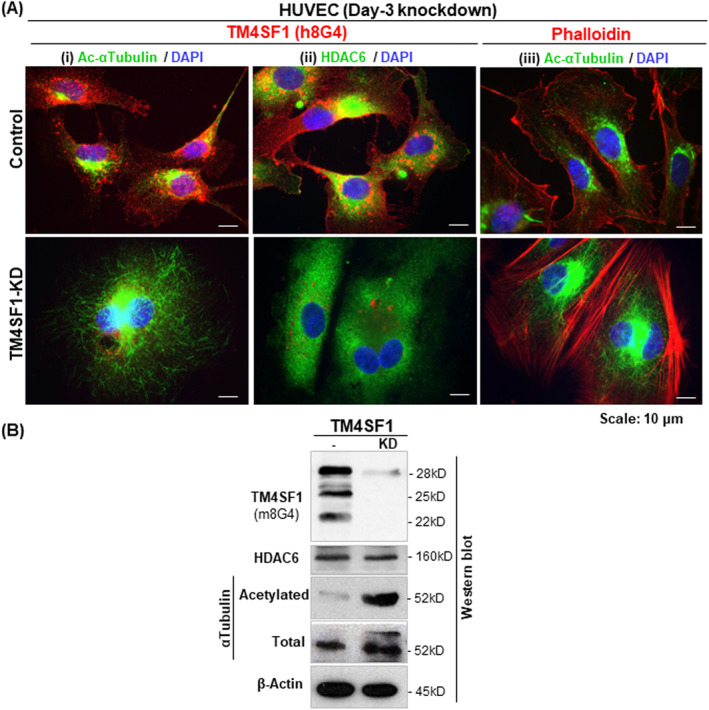
TM4SF1 knockdown leads to extensive microtubule acetylation in HUVEC. (A) Representative immunocytochemical images reveal that HUVECs became enlarged with frequent binuclear morphology that contained extensive stress fiber (iii) and acetylated αTubulin (Lys40) (i,iii), despite an apparently normal level of HDAC6 (ii). (B) Representative Western blot shows that 3‐day TM4SF1‐KD led to greatly reduced levels of all three TM4SF1 protein bands (22‐, 25‐, and 28‐kD) along with a highly increased level of acetylated αTubulin. HDAC6 protein was observed at a similar level before and after TM4SF1‐KD in HUVEC. *β*‐Actin was employed as a protein loading normalizer. HUVEC, human umbilical vein endothelial cells.

HDAC6 has recently emerged as a tubulin deacetylase^8^, ^9^ and chemical inhibition^21^ or silencing^22^ of HDAC6 blocks endothelial cell growth and sprouting. Having shown that HDAC6 associates with and internalizes along with TMED, and having previously shown that HUVEC are unable to polarize for movement or to perform cytokinesis after TM4SF1 knockdown,^7^ we investigated microtubule acetylation status after TM4SF1 knockdown through immunofluorescence staining (Figure 4A) and Western blot analysis (Figure 4B) in control and TM4SF1 knockdown HUVECs. Representative immunofluorescence staining results with an antibody that specifically detects endogenous levels of αTubulin when acetylated at Lys40 showed a substantially greater degree of microtubule acetylation in the TM4SF1‐knockdown HUVECs in comparison to control HUVECs (Figure 4A,i,iii), despite apparently normal HDAC6 protein levels (Figure 4A,ii). Western blot provided supporting evidence of increased levels of acetylated αTubulin when TM4SF1 was depleted through knockdown in HUVEC, though total HDAC6 protein levels were unaffected in comparison to controls (Figure 4B). Our study indicates that in HUVEC, HDAC6 executes its microtubule deacetylation activities by being recruited to TMED.

## 4 DISCUSSION

This study validates our hypothesis that TMED (TM4SF1‐enriched microdomains) function as vehicles to recruit and distribute cargo molecules to intracellular destinations as they internalize along microtubules from the cell surface to the intracellular space. The internalization pathway of TMED is illustrated in Supplementary Figure S1.^8^ Signaling molecules recruited to and transported by TMED include several with functional roles in endothelial cell proliferation and migration, notably PLCγ and HDAC6. When TMED formation is inhibited due to lowered TM4SF1 expression—as following TM4SF1‐knockdown in cultured endothelial cells^7^, ^11^ or in mouse models of VEGFA‐induced angiogenesis in vivo^7^ and in *Tm4sf1*‐null and ‐heterozygous mice^10^ along with results revealed in this study (Figure 1)—endothelial cell function is impaired.

Vasculogenesis and angiogenesis are essential for both physiological and pathologicasl processes that include embryonic development, tissue regeneration, wound healing and tumor growth and metastasis.^23^, ^24^ Apart from its over‐expression in the tumor cells of solid tumors,^2^, ^4^, ^5^, ^7^ high TM4SF1 expression is largely confined to proliferating endothelial and mesenchymal stem cells^9^, ^10^; in vivo, TM4SF1 is required for blood vessel formation and maturation.^7^, ^10^ Indeed, *Tm4sf1*‐null mice are lethal at embryonic day‐9.5, demonstrating an avascular phenotype.^10^ *Tm4SF1*‐heterozygous embryos are smaller in body size during early embryonic development and almost half die in utero due to intracranial hemorrhage; the remaining half are born alive without apparent physiological defects.^10^ In this study, we investigated the ability of live‐born *Tm4sf1*‐heterozygous mice to manage angiogenesis in the challenges of tumor growth and wound healing.

B16F10 tumor growth was substantially delayed in *Tm4sf1*‐heterozygous mice, requiring two weeks longer than in wild‐type mice to grow to 10% of body weight (Figure 1A). Similarly, ear wound closure was considerably impaired, reaching 9%–15% closure in *Tm4sf1*‐heterozygous mice in the first 21 days, compared to 50%–70% closure in wild type littermates (Figure 1B). In both models, the differences between *Tm4sf1*‐wild type and ‐heterozygous mice were seen within the first 2 weeks after the need for angiogenesis was initiated. This indicates that *Tm4sf1*‐heterozygous mice encountered an immediate incompetency in their ability to support angiogenesis under pathological assault in comparison to wild type mice. B16F10 mouse melanoma tumor cells express high levels of TM4SF1 and we have noted reduced cell growth, proliferation and migration when TM4SF1 expression was knocked down in cultures in vitro (unpublished data). Similar observations have been reported in human tumor cell lines.^25^, ^26^ Whether any paracrine mechanism exists in which the reduced TM4SF1 levels in the endothelium of TM4SF1 heterozygotes can influence TM4SF1 expression in tumor cells and consequently affect tumor growth indirectly is currently unknown.

Both tumor growth and wound healing are complex and dynamic processes that are supported by myriad cellular events such as endothelial cell migration and proliferation, their recruitment of differentiating mesenchymal stem cells, and ancillary support from the soluble factors released by the infiltrating leukocytes.^27^, ^28^, ^29^ Given that *Tm4sf1*‐heterozygous mice can manage normal physiological postnatal growth, but are hindered in the performance of angiogenesis under pathological challenge, a natural inference is that timely expression of optimal levels of TM4SF1 to support TM4SF1's critical role in pathology.

A unique function of tetraspanins is their ability to interact with other proteins on the cell membrane by means of their extracellular, transmembrane and cytoplasmic domains, thus permitting them to incorporate multiple proteins into clusters and form tetraspanin‐enriched microdomains.^12^, ^13^ Some tetraspanins, including CD63 and CD105, have been reported to internalize via clathrin‐mediated endocytosis with an ultimate destination being endosomes‐lysosomes or exosomes.^12^, ^13^ Through endocytic membrane vesicles, genuine tetraspanins traffic a wide range of cargo molecules from the cell surface to the interior.^13^, ^14^ Although TM4SF1 is not a member of the genuine tetraspanin family, it does form microdomains like the genuine tetraspanins.^16^ However, TM4SF1 lacks motifs recognized by clathrin adapter proteins^13^, ^14^ and is distinguished by its unique internalization via microtubules with its ultimate destination being the nucleus.^8^

TM4SF1‐deficient endothelial cells lose the ability to grow, proliferate and migrate, functions necessary for angiogenesis.^7^, ^10^, ^11^ We therefore asked whether TMEDs acted in angiogenesis by distributing cargo molecules known to be essential for angiogenesis from the cell surface to the cell interior spaces. The 18 proteins studied here are all positioned downstream of the RTK signaling cascade,^17^, ^18^ and are known to be involved in endothelial cell proliferation (PI4K, PLCγ1, PKCα, MEK1/2, and ERK), migration (αTubulin, HDAC6, IQGAP1, Rac1, Akt, β‐actin, PTEN, PI3Kα,β,p85 and PDK1), nuclear entry (Importin‐β), and endocytosis (clathrin) (Figure 2A). IP‐Westerns revealed that 12 molecules (underlined above) were pulled‐down along with TMED from the endothelial cell surface (Figure 2B). With the exception of PKCα and PI4K, the remaining 10 signaling molecules remained in association with TMED after 2 hours of further culture, when internalization was well underway (Figure 2B). Two signaling molecules, PLCγ1 and HDAC6, caught our immediate attention because PLCγ1 is critically involved in endothelial cell proliferation ^19^ and HDAC6 governs cell migration and apoptosis.^30^, ^31^

VEGFR2 Tyr^1175^ (Tyr^1173^ in mice) is located at the C‐terminal tail and is the docking site for PLCγ1 that releases PLCγ1 upon conformational change through Tyr^1175^‐phosphorylation upon VEGFR2 activation.^19^ This molecular process is thought to be the principal means of VEGF‐dependent activation of the ERK‐MAPK signaling pathway for endothelial cell proliferation; evidence for this includes the observation that PLCγ1‐deficient mice and mouse embryos harboring a knock‐in mutant Flk1 Y1173F allele^32^ phenocopied vasculogenic and hematopoietic lethal defects seen in *Flk1* null mice.^33^ VEGFR2 does not associate with TMED (Figure 3), indicating that the recruitment of PLCγ1 to TMED likely happened after it was released from VEGFR2. This raises the possibility that PLCγ1 downstream signaling, such as its catalytic X/Y domain unmasking for PIP_2_ (phosphatidylinositol‐4,5‐biphosphate) lipid binding and subsequent hydrolysis for the generation of the second messengers DAG (diacylglycerol) and IP_3_ (inositol‐1,4,5‐triphosphate) and their respective activation of cPKC‐MEK‐ERK for cell proliferation and of NFAT for endoplasmic reticulum calcium release,^34^ is achieved through TMED internalization. As TM4SF1 knockdown does not affect VEGFR2 protein levels (Figure 3), the inability of endothelial cells to proliferate in TM4SF1 knockdown cells presumably reflects a disruption of VEGFR2's downstream signaling.

PKCα and PKCβ are the two principal conventional Ca^2+^‐dependent PKC isoforms in endothelial cells^35^ and produce cascade activation of MEK and ERK for the regulation of cell proliferation.^36^ PKCα, MEK, and ERK are all found in association with TMED, although the PKCα interaction appears to be limited to the cell surface while MEK and ERK are internalized along with TMED (Figure 2B,ii). We have not yet examined whether PKCβ and NFAT are also recruited to TMED, nor have we investigated the activation status of those recruited molecules, the kinetics of the recruitment after being treated with a specific growth factor, whether they are interacting with TM4SF1 directly or indirectly through recruited membrane proteins and regulate TM4SF1's nuclear entry. In the current study, we utilized cells grown in complete media which were constantly exposed to four growth factors (VEGFA, bFGF, IGF1, and EGF).

Interestingly, we did not see a direct association of PI3K and PDK1 with TMED, suggesting a limited role of TMED in PIP_3_ (phosphatidylinositol‐3,4,5‐triphosphate) lipid, a PI3K‐catalytic product^37^, ^38^ that recruits PDK1. Nevertheless, Akt, a known substrate of PI3K activation that is involved in cell migration regulation,^39^ along with IQGAP1 and Rac1 (Figure 2B,iii), are recruited to TMED, suggesting that some PI3K downstream mediators influence endothelial cell migration through TMED.

HDAC6 is a microtubule‐associated deacetylase that has been implicated in regulating microtubule stability and function via *α*Tubulin deacetylation.^30^, ^31^ HDAC6 appears to exert its microtubule deacetylation activity through the recruitment to TMED and internalization along microtubules in endothelial cells (Figure 2B,iii), thus causing endothelial cells to retain highly acetylated microtubules although HDAC6 protein were present in the TM4SF1 depleted cells (Figure 4). HDAC6 is also known to (i) interact with the p150^*glued*^ component of the dynein–dynactin microtubule motor complex^20^ and with importin for shuttling to the nucleus^40^; and to (ii) act as a linker between ubiquitinated proteins and dynein motors to deliver the ubiquitinated proteins along microtubule tracks toward aggresomes to process misfolded proteins.^29^ The cellular location of perinuclear TMED before nuclear entry in cells expressing high levels of TM4SF1 in vitro does resemble that of aggresomes (Figure S2,A,b). However, aggresomes have not been reported in endothelial cells and known aggresome forming cells,^29^ such as leukocytes and fibroblasts, lack TM4SF1 expression.^10^ Interestingly, endothelial growth and sprouting activities are shown to be influenced by HDAC6 activity, as evidenced by chemical inhibition^21^ or silencing^22^ of Histone deacetylase (HDAC), suggesting a potential role of HDAC6 in the regulation of TMED internalization, a prospect of which we are currently investigating. HDACs constitute a family of 18 protein members that are highly conserved across all eukaryotes; some localize to the nucleus including HDAC8.^41^ Whether TM4SF1 recruits other members of HDAC family besides HDAC6, and endows them with the ability to localize to the nucleus in endothelial cells, is an issue deserves further investigation.

In conclusion, our studies indicate that TMED recruitment of cargo proteins generates a signaling web that gathers a diverse array of signaling molecules on the cell surface and delivers them to intracellular locations. Cell volume for asynchronously dividing endothelial cells is measured at 882 ± 234 to 1835 ± 282 μm^3^.^42^ Meanwhile, cells with characteristic volumes of 2000–4000 μm^3^ typically retain about 10^10^ protein molecules per cell (2‐4 x10^6^ protein/μm^3^).^43^ Given this crowded nature of intracellular compartments, active molecule trafficking processes, such as that provided by TMED‐mediated cargo distribution, are likely needed to maintain normal cellular function. Advanced techniques, such as mass spectrometry and high‐resolution fluorescence and electron microscopy along with the spatial imaging, are currently planned to address (i) the mechanism of TMED formation, (ii) the identity and activation status of TMED recruited proteins, (iii) their intracellular distribution and spatial cellular relationship to TMED, and (iv) molecular and cellular events influenced by TM4SF1 expression levels in mice in vivo. Ultimately, such studies will elucidate the role of TM4SF1's cargo distribution process in blood vessel formation in normal and under pathological assault in vivo.

## AUTHOR CONTRIBUTIONS

Conceptualization: Chi‐Iou Lin, Anne Merley, Shou‐Ching S. Jaminet; Methodology: Chi‐Iou Lin, Anne Merley, Shou‐Ching S. Jaminet; Validation: Chi‐Iou Lin, Anne Merley; Formal analysis: Chi‐Iou Lin, Anne Merley, Shou‐Ching S. Jaminet; Investigation: Chi‐Iou Lin, Anne Merley, Shou‐Ching S. Jaminet; Resources: Chi‐Iou Lin, Anne Merley, Shou‐Ching S. Jaminet; Data curation: Chi‐Iou Lin, Anne Merley, Shou‐Ching S. Jaminet; Writing ‐ original draft: Shou‐Ching S. Jaminet; Writing ‐ review & editing: Chi‐Iou Lin, Anne Merley, Harold F. Dvorak, Shou‐Ching S. Jaminet; Visualization: Chi‐Iou Lin, Anne Merley, Harold F. Dvorak, Shou‐Ching S. Jaminet; Supervision: Shou‐Ching S. Jaminet; Project administration: Shou‐Ching S. Jaminet; Funding acquisition: Shou‐Ching S. Jaminet, Harold F. Dvorak.

## CONFLICT OF INTEREST STATEMENT

The authors declare no competing or financial interests, or personal relationships that could have appeared to influence the work reported in this paper.

## ETHICS STATEMENT

Studies involving animal experiments were performed under protocols approved by the Animal Care and Use Committee of Beth Israel Deaconess Medical Center.

## Supporting information

Supporting Information S1

Figure S1

Figure S2

Table S1

## Data Availability

All relevant data in this study are included in this published article and its supplementary files.

## References

[1] Wright, M. D. , G. B. Rudy , and J. Ni . 2000. “The L6 Membrane Proteins‐‐a New Four‐Transmembrane Superfamily.” Protein Science 9(8): 1594–1600. 10.1110/ps.9.8.1594. 10975581 PMC2144728

[2] Hellstrom, I. , D. Horn , P. Linsley , J. P. Brown , V. Brankovan , and K. E. Hellstrom . 1986. “Monoclonal Mouse Antibodies Raised against Human Lung Carcinoma.” Cancer Research 46: 3917–3923. 3731064

[3] Denardo, S. J. , L. F. O'Grady , D. J. Macey , L. A. Kroger , G. L. DeNardo , Kathleen R. Lamborn , N. B. Levy , S. L. Mills , I. Hellstrom , and K. E. Hellstrom . 1991. “Quantitative Imaging of Mouse L‐6 Monoclonal Antibody in Breast Cancer Patients to Develop a Therapeutic Strategy.” Int J Rad Appl Instrum B 18(6): 621–631. 10.1016/0883-2897(91)90032-g. 1743985

[4] Denardo, S. J. , G. R. Mirick , L. A. Kroger , L. F. O'Grady , K. L. Erickson , A. Yuan , K. R. Lamborn , I. Hellstrom , K. E. Hellstrom , and G. L. Denardo . 1994. “The Biologic Window for Chimeric L6 Radioimmunotherapy.” Cancer 73(S3): 1023–1032. 10.1002/1097-0142(19940201)73:3+<1023::aid-cncr2820731341>3.0.co;2-u. 8306244

[5] DeNardo, S. J. , and G. L. DeNardo . 2006. “Targeted Radionuclide Therapy for Solid Tumors: an Overview.” International Journal of Radiation Oncology, Biology, Physics 66(2): S89–S95. 10.1016/j.ijrobp.2006.03.066. 16979448

[6] English, S. B. , S.‐C. Shih , M. F. Ramoni , L. E. Smith , and A. J. Butte . 2009. “Use of Bayesian Networks to Probabilistically Model and Improve the Likelihood of Validation of Microarray Findings by RT‐PCR.” J Biomed Inform 42(2): 287–295. 10.1016/j.jbi.2008.08.009. 18790084 PMC3962641

[7] Shih, S.‐C. , A. Zukauskas , D. Li , G. Liu , L.‐H. Ang , J. A. Nagy , L. F. Brown , and H. F. Dvorak . 2009. “The L6 Protein TM4SF1 Is Critical for Endothelial Cell Function and Tumor Angiogenesis.” Cancer Research 69(8): 3272–3277. 10.1158/0008-5472.CAN-08-4886. 19351819 PMC2774109

[8] Sciuto, T. E. , A. Merley , C.‐I. Lin , D. Richardson , Y. Liu , D. Li , A. M. Dvorak , H. F. Dvorak , and S.‐C. S. Jaminet . 2015. “Intracellular Distribution of TM4SF1 and Internalization of TM4SF1‐Antibody Complex in Vascular Endothelial Cells.” Biochemical and Biophysical Research Communications 465(3): 338–343. 10.1016/j.bbrc.2015.07.142. 26241677 PMC4579096

[9] Lin, C.‐I. , A. Merley , T. E. Sciuto , D. Li , A. M. Dvorak , J. M. Melero‐Martin , H. F. Dvorak , and S.‐C. S. Jaminet . 2014. “TM4SF1: a New Vascular Therapeutic Target in Cancer.” Angiogenesis 17(4): 897–907. 10.1007/s10456-014-9437-2. 24986520 PMC4177288

[10] Lin, C. I. , A. Merley , H. Wada , and S. C. Jaminet . 2023. “TM4SF1 Is Essential for Embryonic Blood Vessel Development.” Journal of Blood & Lymph 13: 307–317. https://www.hilarispublisher.com/open-access/tm4sf1-is-essential-for-embryonic-blood-vessel-development-102863.html.

[11] Zukauskas, A. , A. Merley , D. Li , L.‐H. Ang , T. E. Sciuto , S. Salman , A. M. Dvorak , H. F. Dvorak , and S.‐C. S. Jaminet . 2011. “TM4SF1: a Tetraspanin‐like Protein Necessary for Nanopodia Formation and Endothelial Cell Migration.” Angiogenesis 14(3): 345–354. 10.1007/s10456-011-9218-0. 21626280 PMC3298766

[12] Stipp, C. S. , T. V. Kolesnikova , and M. E. Hemler . 2003. “Functional Domains in Tetraspanin Proteins.” Trends Biochem Sci 28(2): 106–112. 10.1016/s0968-0004(02)00014-2. 12575999

[13] van Deventer, S. J. , V.‐M. E. Dunlock , and A. B. van Spriel . 2017. “Molecular Interactions Shaping the Tetraspanin Web.” Biochemical Society Transactions 45(3): 741–750. 10.1042/BST20160284. 28620035

[14] Kelly, B. T. , and D. J. Owen . 2011. “Endocytic Sorting of Transmembrane Protein Cargo.” Current Opinion in Cell Biology 23(4): 404–412. 10.1016/j.ceb.2011.03.004. 21450449

[15] Visintin, A. , K. Knowlton , E. Tyminski , C.‐I. Lin , X. Zheng , K. Marquette , S. Jain , L. Tchistiakova , D. Li , C. J. O'Donnell , A. Maderna , X. Cao , R. Dunn , W. B. Snyder , A. K. Abraham , M. Leal , S. Shetty , A. Barry , L. Zawel , A. J. Coyle , H. F. Dvorak , and S.‐C. Jaminet . 2015. “Novel Anti‐tm4sf1 Antibody‐Drug Conjugates with Activity against Tumor Cells and Tumor Vasculature.” Mol Cancer Ther 14(8): 1868–1876. 10.1158/1535-7163.MCT-15-0188. 26089370

[16] Lin, C.‐I. , C.‐Y. Lau , D. Li , and S.‐C. Jaminet . 2014. “Nanopodia‐‐thin, Fragile Membrane Projections with Roles in Cell Movement and Intercellular Interactions.” Journal of Visualized Experiments: JoVE(86). 10.3791/51320. PMC4161280 24747485

[17] Koch, S. , and L. Claesson‐Welsh . 2012. “Signal Transduction by Vascular Endothelial Growth Factor Receptors.” Cold Spring Harbor perspectives in medicine 2(7): a006502. 10.1101/cshperspect.a006502. 22762016 PMC3385940

[18] Jeltsch, M. , V. M. Leppanen , P. Saharinen , and K. Alitalo . 2013. “Receptor Tyrosine Kinase‐Mediated Angiogenesis.” Cold Spring Harbor Perspectives in Biology 5(9): a009183. 10.1101/cshperspect.a009183. 24003209 PMC3753715

[19] Chen, D. , and M. Simons . 2021. “Emerging Roles of PLCgamma1 in Endothelial Biology.” Science Signaling 14(694). 10.1126/scisignal.abc6612. PMC8507396 34344833

[20] Hubbert, C. , A. Guardiola , R. Shao , Y. Kawaguchi , A. Ito , A. Nixon , M. Yoshida , X.‐F. Wang , and T.‐P. Yao . 2002. “HDAC6 Is a Microtubule‐Associated Deacetylase.” Nature 417(6887): 455–458. 10.1038/417455a. 12024216

[21] Nomura, Y. , M. Nakano , H. Woo Sung , M. Han , and D. Pandey . 2021. “Inhibition of HDAC6 Activity Protects against Endothelial Dysfunction and Atherogenesis In Vivo: A Role for HDAC6 Neddylation.” Frontiers in Physiology 12: 675724. 10.3389/fphys.2021.675724. 34220539 PMC8245780

[22] Kaluza, D. , J. Kroll , S. Gesierich , T.‐P. Yao , R. A. Boon , E. Hergenreider , Marc Tjwa , L. Rössig , E. Seto , H. G. Augustin , A. M. Zeiher , S. Dimmeler , and C. Urbich . 2011. “Class IIb HDAC6 Regulates Endothelial Cell Migration and Angiogenesis by Deacetylation of Cortactin.” EMBO J 30(20): 4142–4156. 10.1038/emboj.2011.298. 21847094 PMC3199386

[23] Kubis, N. , and B. I. Levy . 2003. “Vasculogenesis and Angiogenesis: Molecular and Cellular Controls. Part 1: Growth Factors.” Interv Neuroradiol 9(3): 227–237. 10.1177/159101990300900301. 20591248 PMC3548208

[24] Dvorak, H. F. 2019. “Tumors: Wounds that Do Not Heal‐A Historical Perspective with a Focus on the Fundamental Roles of Increased Vascular Permeability and Clotting.” Seminars in Thrombosis and Hemostasis 45(06): 576–592. 10.1055/s-0039-1687908. 31096305

[25] Sun, Y. , Y. Xu , J. Xu , D. Lu , and J. Wang . 2015. “Role of TM4SF1 in Regulating Breast Cancer Cell Migration and Apoptosis through PI3K/AKT/mTOR Pathway.” International Journal of Clinical and Experimental Pathology 8: 9081–9088. 26464650 PMC4583882

[26] Cao, J. , J.‐C. Yang , V. Ramachandran , T. Arumugam , D.‐F. Deng , Z.‐S. Li , L.‐M. Xu , and C. D. Logsdon . 2016. “TM4SF1 Regulates Pancreatic Cancer Migration and Invasion In Vitro and In Vivo.” Cellular Physiology and Biochemistry 39(2): 740–750. 10.1159/000445664. 27459514

[27] Ding, R. , D. C. Darland , M. S. Parmacek , and P. A. D'Amore . 2004. “Endothelial‐mesenchymal Interactions In Vitro Reveal Molecular Mechanisms of Smooth Muscle/pericyte Differentiation.” Stem Cells Dev 13(5): 509–520. 10.1089/1547328042417336. 15588508

[28] Nagy, J. A. , A. M. Dvorak , and H. F. Dvorak . 2007. “VEGF‐A and the Induction of Pathological Angiogenesis.” Annual Review of Pathology: Mechanisms of Disease 2(1): 251–275. 10.1146/annurev.pathol.2.010506.134925. 18039100

[29] Carmona, B. , H. S. Marinho , C. L. Matos , S. Nolasco , and H. Soares . 2023. “Tubulin Post‐Translational Modifications: The Elusive Roles of Acetylation.” Biology 12(4): 561. 10.3390/biology12040561. 37106761 PMC10136095

[30] Gudimchuk, N. B. , and J. R. McIntosh . 2021. “Regulation of Microtubule Dynamics, Mechanics and Function through the Growing Tip.” Nature Reviews Molecular Cell Biology 22(12): 777–795. 10.1038/s41580-021-00399-x. 34408299

[31] Li, L. , and X.‐J. Yang . 2015. “Tubulin Acetylation: Responsible Enzymes, Biological Functions and Human Diseases.” Cellular and Molecular Life Sciences 72(22): 4237–4255. 10.1007/s00018-015-2000-5. 26227334 PMC11113413

[32] Takahashi, T. , S. Yamaguchi , K. Chida , and M. Shibuya . 2001. “A Single Autophosphorylation Site on KDR/Flk‐1 Is Essential for VEGF‐A‐dependent Activation of PLC‐Gamma and DNA Synthesis in Vascular Endothelial Cells.” Embo J 20(11): 2768–2778. 10.1093/emboj/20.11.2768. 11387210 PMC125481

[33] Sakurai, Y. , K. Ohgimoto , Y. Kataoka , N. Yoshida , and M. Shibuya . 2005. “Essential Role of Flk‐1 (VEGF Receptor 2) Tyrosine Residue 1173 in Vasculogenesis in Mice.” Proc Natl Acad Sci U S A 102(4): 1076–1081. 10.1073/pnas.0404984102. 15644447 PMC545830

[34] Nakamura, Y. , and K. Fukami . 2017. “Regulation and Physiological Functions of Mammalian Phospholipase C.” Journal of biochemistry 161: 315–321. 10.1093/jb/mvw094. 28130414

[35] Xia, P. , L. P. Aiello , H. Ishii , Z. Y. Jiang , D. J. Park , G. S. Robinson , H. Takagi , W. P. Newsome , M. R. Jirousek , and G. L. King . 1996. “Characterization of Vascular Endothelial Growth Factor's Effect on the Activation of Protein Kinase C, its Isoforms, and Endothelial Cell Growth.” Journal of Clinical Investigation 98(9): 2018–2026. 10.1172/JCI119006. 8903320 PMC507645

[36] Mackay, H. J. , and C. J. Twelves . 2007. “Targeting the Protein Kinase C Family: Are We There yet?” Nature Reviews Cancer 7: 554–562. 10.1038/nrc2168. 17585335

[37] Raimondi, C. , A. Chikh , T. Maffucci , M. Falasca , and M. Falasca . 2012. “A Novel Regulatory Mechanism Links PLCgamma1 to PDK1.” Journal of Cell Science 125: 3153–3163. 10.1242/jcs.100511. 22454520 PMC3434861

[38] Raimondi, C. , V. Calleja , R. Ferro , A. Fantin , A. M. Riley , B. V. L. Potter , C. H. Brennan , T. Maffucci , B. Larijani , and M. Falasca . 2016. “A Small Molecule Inhibitor of PDK1/PLCgamma1 Interaction Blocks Breast and Melanoma Cancer Cell Invasion.” Scientific Reports 6(1): 26142. 10.1038/srep26142. 27199173 PMC4873738

[39] He, Y. , M. M. Sun , G. G. Zhang , J. Yang , K. S. Chen , W. W. Xu , and B. Li . 2021. “Targeting PI3K/Akt Signal Transduction for Cancer Therapy.” Signal Transduct Target Ther 6(1): 425. 10.1038/s41392-021-00828-5. 34916492 PMC8677728

[40] Liu, Y. , L. Peng , E. Seto , S. Huang , and Y. Qiu . 2012. “Modulation of Histone Deacetylase 6 (HDAC6) Nuclear Import and Tubulin Deacetylase Activity through Acetylation.” Journal of Biological Chemistry 287(34): 29168–29174. 10.1074/jbc.M112.371120. 22778253 PMC3436516

[41] Milazzo, G. , D. Mercatelli , G. Di Muzio , L. Triboli , P. De Rosa , G. Perini , and F. M. Giorgi . 2020. “Histone Deacetylases (HDACs): Evolution, Specificity, Role in Transcriptional Complexes, and Pharmacological Actionability.” Genes 11(5): 556. 10.3390/genes11050556. 32429325 PMC7288346

[42] Rubin, D. B. , E. A. Drab , and K. D. Bauer . 1989. “Endothelial Cell Subpopulations In Vitro: Cell Volume, Cell Cycle, and Radiosensitivity.” Journal of Applied Physiology 67(4): 1585–1590. 10.1152/jappl.1989.67.4.1585. 2793759

[43] Milo, R. 2013. “What Is the Total Number of Protein Molecules Per Cell Volume? A Call to Rethink Some Published Values.” BioEssays 35(12): 1050–1055. 10.1002/bies.201300066. 24114984 PMC3910158

