# Supplementary material for: TM4SF1 is a molecular facilitator that distributes cargo proteins intracellularly in endothelial cells in support of blood vessel formation
Source: J Cell Commun Signal. 2024 May 7;18(2):e12031. doi: 10.1002/ccs3.12031 (PMC11208120; doi:10.1002/ccs3.12031)
Supplement: Supplementary file 1 — Supporting Information S1 [file CCS3-18-e12031-s004.docx]

**Supplementary Figure Legends**

**Supplementary Figure 1. TM4SF1-enriched microdomain (TMED) internalization.** TMEDs are transported along microtubules to the microtubule organization center (MTOC) and then enter the nucleus via nuclear pores where TMED are being processed.

**Supplementary Figure 2. TM4SF1 protein characteristics in HUVEC**. HUVEC were harvested in suspension and performed flow cytometry **(A,a)**, immunocytochemistry **(A.b)**, Western blot **(B)**, and immunoprecipitation (IP)-Western blot **(C)**. **(A,a)** HUVECs showed 100% positivity with h8G4-Alexa488 (Alexa488-directly conjugated anti-human TM4SF1 antibody 8G4 with human IgG1 constant region) in flow cytometry detection in TBS without Triton X-100 treatment. The bound antibody fluorescence intensities dropped to 12.7% and 0.5% after the 0.05% and 0.1% Triton X-100 treatments, respectively. hIgG1 with 2^nd^ anti-human IgG-Alexa488 combination did not interact with any proteins on HUVEC in flow cytometry and showed a background fluorescence intensity of 0.5%. **(A,b,i)** Positively stained TMED appeared throughout the cell surface (yellow arrows) with intense congregated microdomains in the perinuclear region (white arrows) without Triton treatment; **(A,b,ii)** 0.05% Triton/TBS treatment removed most of the TMED and left behind some residual microdomains in the perinuclear region (white arrows); **(iii)** 0.1% Triton/TBS treatment removed TMED including the residual signals from the perinuclear region. **(B)** HUVECs in suspension were sequentially incubated in a TBS buffer containing 0%, 0.05% or 0.1% Triton to extract proteins for Western blot. m8G4 (anti-human TM4SF1 antibody 8G4 with mouse IgG1 constant region) identified all three (22kD, 25kD, and 28kD) TM4SF1 protein bands in the 0.05% Triton extract; the longer exposure time further revealed the low levels of 28kD TM4SF1 protein extract through 0.1% Triton. **(C)** IP extract contained mostly 28kD TM4SF1 with some residual 25kD TM4SF1. Antibodies against phosphotyrosine (Tyr-P; 4G10) showed positive interactions with the 28kD TM4SF1. A 2^nd^ Ab against the heavy chain (HC) of the antibodies used in IP was applied for monitoring experiments and sample loading in SDS-PAGE. Lack of hIgG1 binding to HUVEC led to HC detection in Western blot.
