# Supplementary figures and images for: TM4SF1 is a molecular facilitator that distributes cargo proteins intracellularly in endothelial cells in support of blood vessel formation
Source: J Cell Commun Signal. 2024 May 7;18(2):e12031. doi: 10.1002/ccs3.12031 (PMC11208120; doi:10.1002/ccs3.12031)

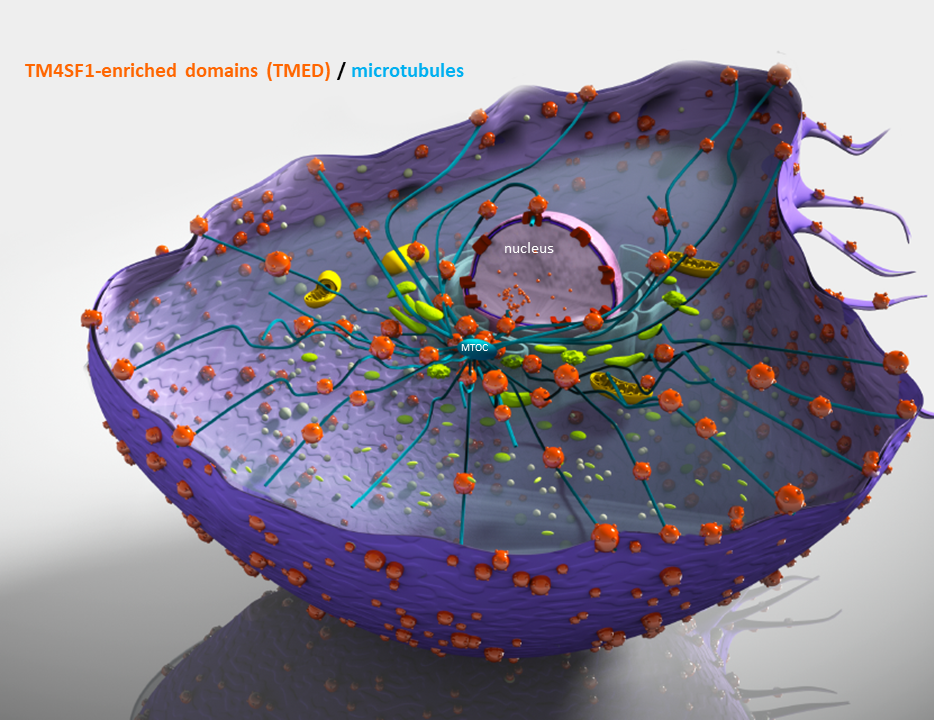

Supplement: Supplementary file 2 — Figure S1 [file CCS3-18-e12031-s003.tif]

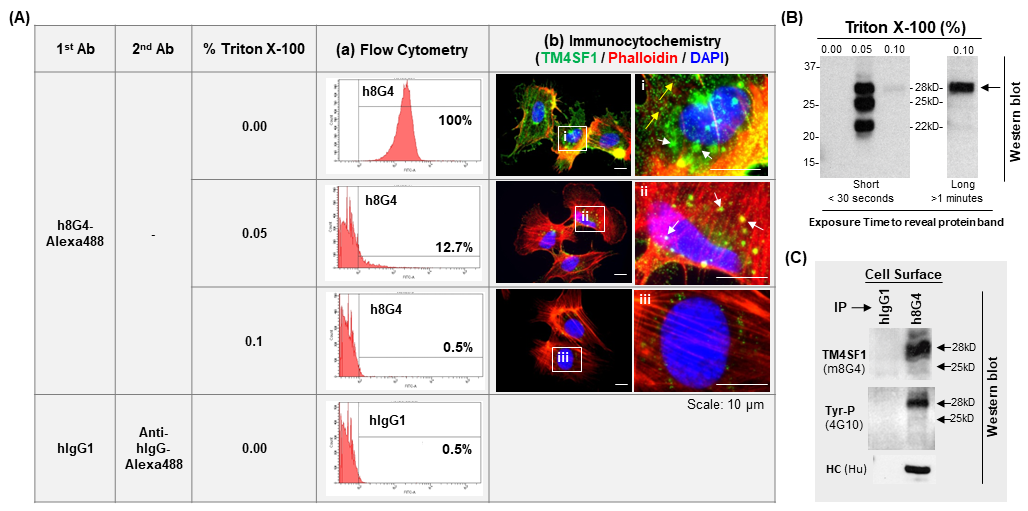

Supplement: Supplementary file 3 — Figure S2 [file CCS3-18-e12031-s002.tif]
