# Supplementary material for: TM4SF1 is a molecular facilitator that distributes cargo proteins intracellularly in endothelial cells in support of blood vessel formation
Source: J Cell Commun Signal. 2024 May 7;18(2):e12031. doi: 10.1002/ccs3.12031 (PMC11208120; doi:10.1002/ccs3.12031)
Supplement: Supplementary file 4 — Table S1 [file CCS3-18-e12031-s001.pptx]

## Slide 1
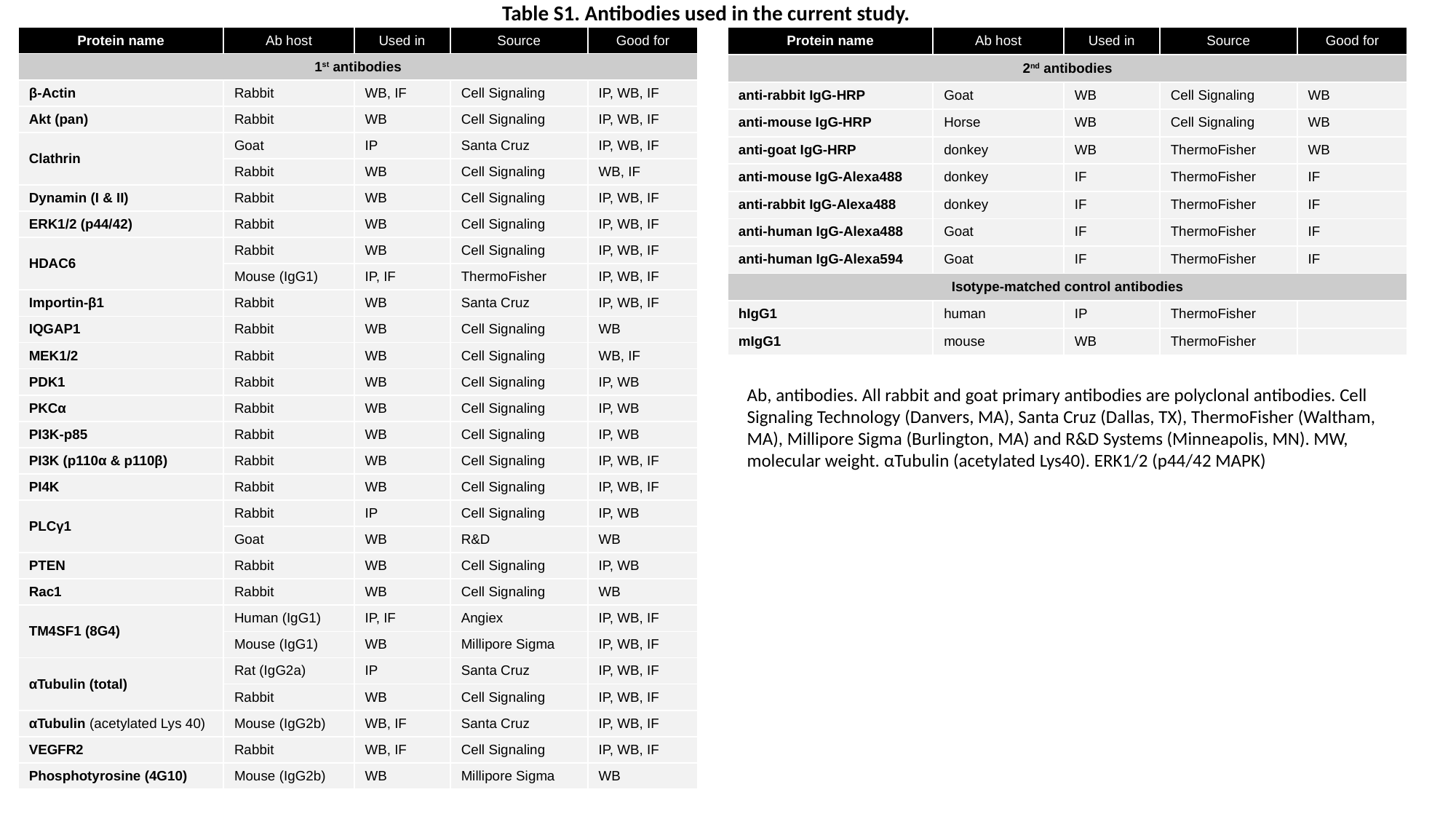

Table S1. Antibodies used in the current study.
| Protein name | Ab host | Used in | Source | Good for |
| --- | --- | --- | --- | --- |
| 1st antibodies | | | | |
| β-Actin | Rabbit | WB, IF | Cell Signaling | IP, WB, IF |
| Akt (pan) | Rabbit | WB | Cell Signaling | IP, WB, IF |
| Clathrin | Goat | IP | Santa Cruz | IP, WB, IF |
| | Rabbit | WB | Cell Signaling | WB, IF |
| Dynamin (I & II) | Rabbit | WB | Cell Signaling | IP, WB, IF |
| ERK1/2 (p44/42) | Rabbit | WB | Cell Signaling | IP, WB, IF |
| HDAC6 | Rabbit | WB | Cell Signaling | IP, WB, IF |
| | Mouse (IgG1) | IP, IF | ThermoFisher | IP, WB, IF |
| Importin-β1 | Rabbit | WB | Santa Cruz | IP, WB, IF |
| IQGAP1 | Rabbit | WB | Cell Signaling | WB |
| MEK1/2 | Rabbit | WB | Cell Signaling | WB, IF |
| PDK1 | Rabbit | WB | Cell Signaling | IP, WB |
| PKCα | Rabbit | WB | Cell Signaling | IP, WB |
| PI3K-p85 | Rabbit | WB | Cell Signaling | IP, WB |
| PI3K (p110α & p110β) | Rabbit | WB | Cell Signaling | IP, WB, IF |
| PI4K | Rabbit | WB | Cell Signaling | IP, WB, IF |
| PLCγ1 | Rabbit | IP | Cell Signaling | IP, WB |
| | Goat | WB | R&D | WB |
| PTEN | Rabbit | WB | Cell Signaling | IP, WB |
| Rac1 | Rabbit | WB | Cell Signaling | WB |
| TM4SF1 (8G4) | Human (IgG1) | IP, IF | Angiex | IP, WB, IF |
| | Mouse (IgG1) | WB | Millipore Sigma | IP, WB, IF |
| αTubulin (total) | Rat (IgG2a) | IP | Santa Cruz | IP, WB, IF |
| | Rabbit | WB | Cell Signaling | IP, WB, IF |
| αTubulin (acetylated Lys 40) | Mouse (IgG2b) | WB, IF | Santa Cruz | IP, WB, IF |
| VEGFR2 | Rabbit | WB, IF | Cell Signaling | IP, WB, IF |
| Phosphotyrosine (4G10) | Mouse (IgG2b) | WB | Millipore Sigma | WB |
| Protein name | Ab host | Used in | Source | Good for |
| --- | --- | --- | --- | --- |
| 2nd antibodies | | | | |
| anti-rabbit IgG-HRP | Goat | WB | Cell Signaling | WB |
| anti-mouse IgG-HRP | Horse | WB | Cell Signaling | WB |
| anti-goat IgG-HRP | donkey | WB | ThermoFisher | WB |
| anti-mouse IgG-Alexa488 | donkey | IF | ThermoFisher | IF |
| anti-rabbit IgG-Alexa488 | donkey | IF | ThermoFisher | IF |
| anti-human IgG-Alexa488 | Goat | IF | ThermoFisher | IF |
| anti-human IgG-Alexa594 | Goat | IF | ThermoFisher | IF |
| Isotype-matched control antibodies | | | | |
| hIgG1 | human | IP | ThermoFisher | |
| mIgG1 | mouse | WB | ThermoFisher | |
Ab, antibodies. All rabbit and goat primary antibodies are polyclonal antibodies. Cell Signaling Technology (Danvers, MA), Santa Cruz (Dallas, TX), ThermoFisher (Waltham, MA), Millipore Sigma (Burlington, MA) and R&D Systems (Minneapolis, MN). MW, molecular weight. αTubulin (acetylated Lys40). ERK1/2 (p44/42 MAPK)
